# Pimobendan B from powder diffraction data

**DOI:** 10.1107/S1600536813028353

**Published:** 2013-10-19

**Authors:** Alvis Zvirgzdins, Mara Delina, Anatoly Mishnev, Andris Actins

**Affiliations:** a University of Latvia, Department of Chemistry, Kr. Valdemara Street 48, Riga, LV-1013, Latvia; b Latvian Institute of Organic Synthesis, Aizkraukles Street 21, Riga, LV-1006, Latvia

## Abstract

The title mol­ecule, C_19_H_18_N_4_O_2_ {systematic name: (*RS*)-6-[2-(4-meth­oxy­phen­yl)-1*H*-benzimidazol-5-yl]-5-methyl-4,5-di­hydro­pyridazin-3(2*H*)-one}, adopts an extended conformation. The dihedral angles between the central benzimidazole ring sytem and the pendant meth­oxy­phenyl and pyridazinone residues are 1.41 (18) and 9.7 (3)°, respectively. In the crystal, N—H⋯N hydrogen bonds link the imadazole groups into [001] chains, and pairs of N—H⋯O hydrogen bonds link the pyridazinone groups into dimers. Together, these generate a two-dimensional supra­molecular structure parallel to (010). The layers are linked by C—H⋯π inter­actions.

## Related literature

For general information about pimobendan, see: Gordon *et al.* (2006 ▶). For related crystalline forms, see: Boeren *et al.* (2011 ▶). Semi-empirical calculations were carried out with *HYPERCHEM Professional* (Hypercube, 2010 ▶). Refinement of lattice parameters and peak profile determination were performed by Le Bail profile fitting (Le Bail *et al.*, 1988 ▶)
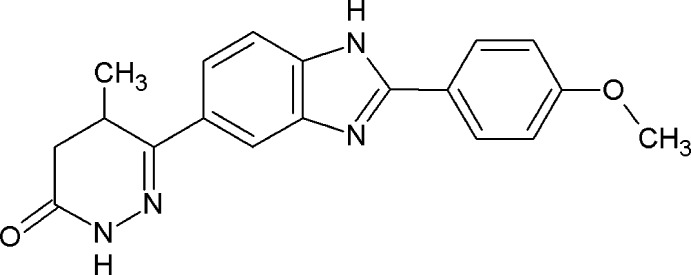


## Experimental

### 

#### Crystal data

C_19_H_18_N_4_O_2_
*M* *_r_* = 334.37
Monoclinic, 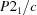

*a* = 18.891 (5) Å
*b* = 9.9619 (5) Å
*c* = 9.5029 (8) Å
β = 90.799 (13)°
*V* = 1788.2 (5) Å^3^
*Z* = 4
Cu *K*α radiation
λ = 1.54184 Å
μ = 0.68 mm^−1^
*T* = 293 K
cylinder, 16 × 0.5 mm

#### Data collection

Bruker D8 diffractometer
Specimen mounting: capillary
Data collection mode: transmission
Scan method: step
2θ_min_ = 3.5°, 2θ_max_ = 70.00°, 2θ_step_ = 0.01°

#### Refinement

*R*_p_ = 0.019
*R*_wp_ = 0.026
*R*_exp_ = 0.020
*R*_Bragg_ = 0.015
χ^2^ = 1.690
6651 data points
134 parameters
56 restraints
H-atom parameters not refined

### 

Data collection: *Dicvol* (Boultif & Louër, 2004 ▶); cell refinement: *FOX* (Favre-Nicolin & Černý, 2002 ▶); data reduction: *FOX*; program(s) used to solve structure: *FOX*; program(s) used to refine structure: *FULLPROF* (Rodriguez-Carvajal, 1993 ▶), *CRYSTALS* (Betteridge *et al.*, 2003 ▶) and *PLATON* (Spek, 2009 ▶); molecular graphics: *Mercury* (Macrae *et al.*, 2008 ▶); software used to prepare material for publication: *WinPlotr* (Roisnel & Rodriguez-Carvajal, 2000 ▶) and *publCIF* (Westrip, 2010 ▶).

## Supplementary Material

Crystal structure: contains datablock(s) global, I. DOI: 10.1107/S1600536813028353/hb7139sup1.cif

Rietveld powder data: contains datablock(s) I. DOI: 10.1107/S1600536813028353/hb7139Isup2.rtv

Supplementary material file. DOI: 10.1107/S1600536813028353/hb7139Isup3.cdx

Supplementary material file. DOI: 10.1107/S1600536813028353/hb7139Isup4.cml

Additional supplementary materials:  crystallographic information; 3D view; checkCIF report

## Figures and Tables

**Table 1:** Hydrogen-bond geometry (Å, °) *Cg*1 is the centroid of the C12/C20/C15/C24/C22/C21 ring.

*D*—H⋯*A*	*D*—H	H⋯*A*	*D*⋯*A*	*D*—H⋯*A*
N3—H42⋯O9^i^	0.97	1.85	2.817 (3)	174
N11—H43⋯N1^ii^	0.95	2.27	3.2039 (19)	168
C18—H26⋯*Cg*1^iii^	0.97	2.43	3.369 (2)	161

Symmetry codes: (i) 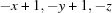; (ii) 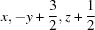; (iii) 
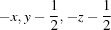
.

**Fig. 1.**
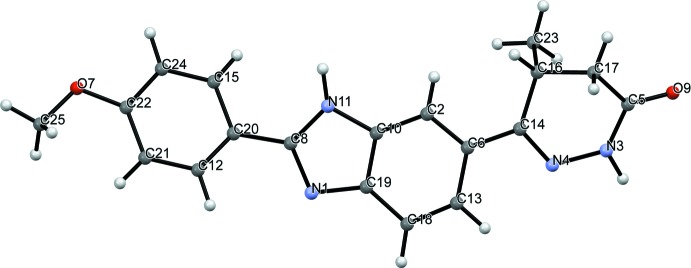
The molecular structure of the title compound showing 50% probability ellipsoids and hydrogen atoms are shown as small spheres of arbitrary radii.

**Fig. 2.**
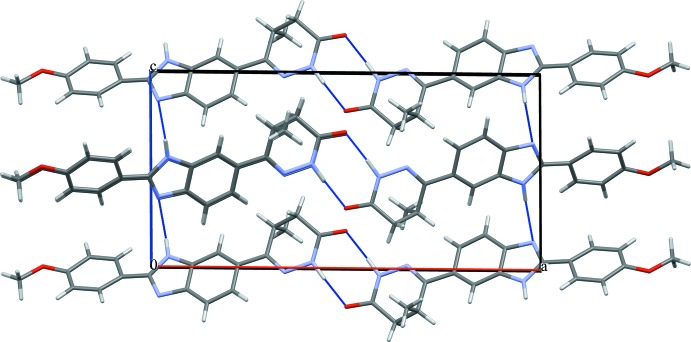
Packing diagram of the title compound viewed along the b axis. Blue lines indicate hydrogen bonds.

**Fig. 3.**
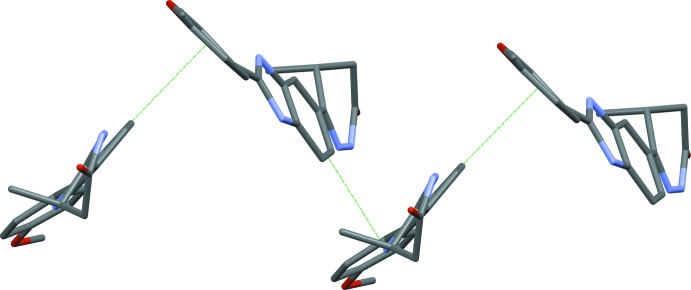
Stacking interactions in the crystal structure of title compound

**Fig. 4.**
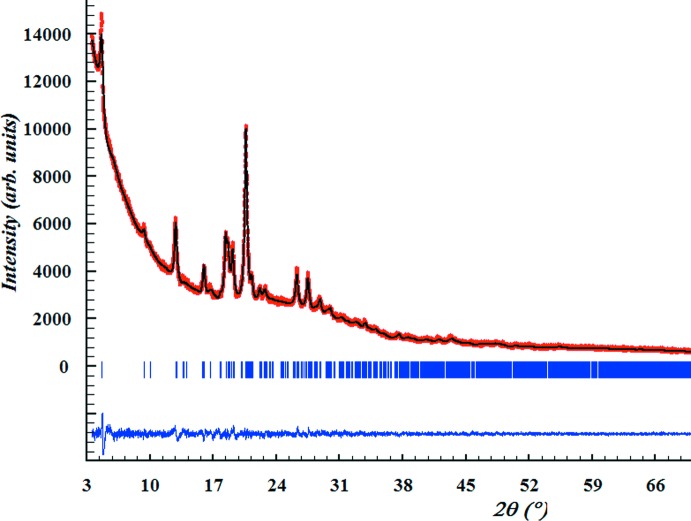
Scattered X-ray intensities of title compound at ambient conditions as a function of diffraction angle 2θ. The observed pattern (red dots), the best Rietveld fit profiles (line) and the difference curve between the observed and calculated profiles (below) are shown.

## supplementary crystallographic information

### 3. Experimental

Indexing of patterns was performed with *WinPlotr* (Roisnel & Rodriguez-Carvajal, 2000) and *Dicvol* (Boultif & Louër, 2004) using reflections in the 2θ range of 3.00 – 30.00°. Space groups for all polymorphs were determined using *FOX 1.9.7.0* (Favre-Nicolin & Černý, 2002). The correct space group was selected based on possible systematic extinctions. The compositions of unit cell and the values of Z were determined in all cases from the unit cell volume.

Refinement of lattice parameters and peak profile determination were performed by Le Bail profile fitting (Le Bail *et al.*, 1988) using *FOX*. Structures were determined with *FOX* by parallel tempering algorithm. The best cost function values were reached by using automatic temperature schedule and Cauchy-type displacement amplitude schedule.

The input model of pimobendan molecule was obtained from semiempirical calculations by *HYPERCHEM Professional* (Hypercube, 2010) for both - R and S enentiomer. The molecules were described in terms of Fenske-Hall Z-matrix format ans structure solutions The dihedral angles C21—C22—O7—C25; N11—C8—C20—C15 and C2—C6—C14—N4 were defined as intra­molecular degrees of freedom and were varied during the structure determinations.

### 3.1. Synthesis and crystallization

Pimobendan form B was prepared in three steps. At the first step, its dioxane solvate was held in a thermostat at 100°C for one day. At the second step obtained powder were suspended in methanol and suspension were hold in a dry box while all methanol evaporates. At the end obtained methanol solvate were desolvatated at 100°C.

### 3.2. Refinement

Rietveld refinement for the final structure was performed by *Fullprof*. Hydrogen atoms were added with *Crystals* according to the molecular geometry and their positions were not refined. Since the bond lengths and angles departed to unacceptable values, atomic parameters for (N3, N4, C5, O9, C14, C16, C17,C23), (N1, N11, C2, C6, C8, C10, C13, C18, C19) and (O7, C12, C15, C20, C21, C22, C24, C25) were refined as rigid bodies.

### 4. Results and discussion

Several crysltalline forms of pimobendan and its preparation are patented (Boeren *et al.*, 2011) but there are no crystal data for these polymorphs or pseudopolymorhs. This article is focused on the structure determination from powder data and description of the pimobendan B form.

Lowest value of cost function were obtained by using molecular model of R enanti­omer in structure determination process. The final structure of pimobendane B form shows that pimobendane molecule is almost linear because the dihedral angle value of N11—C8—C20—C15 = 9.7 (3)° and C13—C6—C14—N4 = 1.41 (18)°. The crystal structure of title compound consist of molecules that are conected via hydrogen bonds that are formed between two imidazole groups (N11—H43···N1^ii^) and two di­hydro­pyradazinone groups (N3—H42···O9^i^ and N3^i^—H42···O9). There are T-shaped C—H···π stacking inter­actions between benzol in meth­oxy­phenyl and benzimidazol groups.

Modeling with PLATON (Spek, 2009) showed that the crystal structure contain voids ( 69Å^3^) accessible to solvent molecules. Since pimobendan B form are obtained from its methanol solvate by desolvation at 100°C, these voids may be result of desolvation at temperature that is almost twice as large as boiling point this solvent. Pimobendan B form at ambient conditions tends to form monohydrate. Unstabilty of pimobendane B form at ambient conditions may be explained by penetration of water molecules into voids of crystal structure.

### Crystal data

**Table:**

C_19_H_18_N_4_O_2_	*V* = 1788.2 (5) Å^3^
*M**_r_* = 334.37	*Z* = 4
Monoclinic, *P*2_1_/*c*	*D*_x_ = 1.24 Mg m^−^^3^
Hall symbol: -P 2ybc	Cu *K*α radiation, λ = 1.54184 Å
*a* = 18.891 (5) Å	µ = 0.68 mm^−^^1^
*b* = 9.9619 (5) Å	*T* = 293 K
*c* = 9.5029 (8) Å	white
β = 90.799 (13)°	cylinder, 16 × 0.5 mm

### Data collection

**Table:**

Bruker D8 diffractometer	Data collection mode: transmission
Radiation source: sealed X-ray tube	Scan method: step
None monochromator	2θ_min_ = 3.5°, 2θ_max_ = 70.00°, 2θ_step_ = 0.01°
Specimen mounting: capillary	

### Refinement

**Table:**

Refinement on *I*_net_	134 parameters
Least-squares matrix: full	56 restraints
*R*_p_ = 0.019	75 constraints
*R*_wp_ = 0.026	Hydrogen site location: inferred from neighbouring sites
*R*_exp_ = 0.020	H-atom parameters not refined
*R*_Bragg_ = 0.015	
χ^2^ = 1.690	(Δ/σ)_max_ = 0.01
6651 data points	Background function: linear extrapolation
Profile function: Pseudo Voigt	

### Special details

**Table:**

Refinement. Rietveld refinement for the final structure was performed by *Fullprof*. Hydrogen atoms were added with *Crystals* according to the molecular geometry and their positions were not refined, but final refinement was performed with hydrogen atoms. Since the bond lengths and angles departed to unacceptable values, atomic parameters for (N3, N4, C5, O9, C14, C16, C17,C23), (N1, N11, C2, C6, C8, C10, C13, C18, C19) and (O7, C12, C15, C20, C21, C22, C24, C25) were refined as rigid bodies.

### Fractional atomic coordinates and isotropic or equivalent isotropic displacement parameters (Å^2^)

**Table:**

	*x*	*y*	*z*	*U*_iso_*/*U*_eq_	
N1	0.01915 (12)	0.62643 (12)	−0.14418 (12)	0.01267*	
C2	0.17167 (12)	0.65440 (12)	0.07706 (12)	0.01267*	
N3	0.41366 (13)	0.52345 (13)	0.03230 (13)	0.01267*	
N4	0.34263 (13)	0.53781 (13)	−0.01834 (13)	0.01267*	
C5	0.43319 (13)	0.53662 (13)	0.17085 (13)	0.01267*	
C6	0.22286 (12)	0.58183 (12)	0.01645 (12)	0.01267*	
O7	−0.27870 (17)	0.93069 (17)	0.00050 (17)	0.01267*	
C8	−0.00564 (12)	0.70860 (12)	−0.04114 (12)	0.01267*	
O9	0.49920 (13)	0.54750 (13)	0.19404 (13)	0.01267*	
C10	0.10535 (12)	0.66256 (12)	0.01531 (12)	0.01267*	
N11	0.04376 (12)	0.73283 (12)	0.05732 (12)	0.01267*	
C12	−0.13383 (17)	0.70975 (17)	−0.11682 (17)	0.01267*	
C13	0.20788 (12)	0.51835 (12)	−0.11605 (12)	0.01267*	
C14	0.29607 (13)	0.58849 (13)	0.06534 (13)	0.01267*	
C15	−0.09123 (17)	0.88148 (17)	0.04048 (17)	0.01267*	
C16	0.31636 (13)	0.63793 (13)	0.21183 (13)	0.01267*	
C17	0.37363 (13)	0.54600 (13)	0.27154 (13)	0.01267*	
C18	0.14285 (12)	0.52292 (12)	−0.18095 (12)	0.01267*	
C19	0.09089 (12)	0.59914 (12)	−0.11154 (12)	0.01267*	
C20	−0.07552 (17)	0.77153 (17)	−0.04901 (17)	0.01267*	
C21	−0.20225 (17)	0.76004 (17)	−0.10811 (17)	0.01267*	
C22	−0.21504 (17)	0.87352 (17)	−0.02608 (17)	0.01267*	
C23	0.34340 (13)	0.78483 (13)	0.19844 (13)	0.01267*	
C24	−0.15891 (17)	0.93136 (17)	0.05152 (17)	0.01267*	
C25	−0.33907 (17)	0.86902 (17)	−0.06608 (17)	0.01267*	
H26	0.13416	0.47697	−0.26987	0.0152*	
H27	0.24355	0.46651	−0.16258	0.0152*	
H28	0.18217	0.69913	0.16336	0.0152*	
H29	−0.05380	0.92376	0.09189	0.0152*	
H30	−0.16806	1.00299	0.11652	0.0152*	
H31	−0.24066	0.72624	−0.16210	0.0152*	
H32	−0.12618	0.63253	−0.17586	0.0152*	
H33	−0.38045	0.92304	−0.04037	0.0152*	
H34	−0.34437	0.78145	−0.02928	0.0152*	
H35	−0.33452	0.86891	−0.16468	0.0152*	
H36	0.27557	0.63704	0.27202	0.0152*	
H37	0.35319	0.45789	0.28346	0.0152*	
H38	0.39052	0.57826	0.36071	0.0152*	
H39	0.35524	0.82003	0.29000	0.0152*	
H40	0.30710	0.84168	0.15603	0.0152*	
H41	0.38474	0.78628	0.14061	0.0152*	
H42	0.44322	0.50470	−0.04847	0.0152*	
H43	0.03860	0.78640	0.13920	0.0152*	

### Geometric parameters (Å, º)

**Table:**

O7—C22	1.358 (4)	C16—C23	1.556 (2)
O7—C25	1.435 (4)	C16—C17	1.521 (3)
O9—C5	1.268 (3)	C18—C19	1.412 (3)
N1—C8	1.3642 (19)	C21—C22	1.396 (2)
N1—C19	1.413 (3)	C22—C24	1.407 (4)
N3—N4	1.426 (3)	C2—H28	0.95
N3—C5	1.3687 (19)	N3—H42	0.97
N4—C14	1.296 (3)	N11—H43	0.95
N11—C8	1.334 (3)	C12—H32	0.96
N11—C10	1.420 (3)	C13—H27	0.96
C2—C6	1.344 (3)	C15—H29	0.95
C2—C10	1.378 (3)	C16—H36	0.97
C5—C17	1.490 (3)	C17—H37	0.97
C6—C14	1.454 (3)	C17—H38	0.96
C6—C13	1.4337 (17)	C18—H26	0.97
C8—C20	1.462 (4)	C21—H31	0.94
C10—C19	1.3848 (17)	C23—H39	0.96
C12—C20	1.410 (4)	C23—H40	0.97
C12—C21	1.390 (4)	C23—H41	0.96
C13—C18	1.368 (3)	C24—H30	0.96
C14—C16	1.5207 (19)	C25—H33	0.98
C15—C24	1.377 (4)	C25—H34	0.95
C15—C20	1.421 (3)	C25—H35	0.94
			
C22—O7—C25	116.05 (17)	C14—C16—C17	108.38 (13)
C8—N1—C19	107.21 (14)	C14—C16—C23	107.98 (10)
N4—N3—C5	123.69 (18)	C17—C16—C23	111.36 (18)
N3—N4—C14	118.38 (14)	C5—C17—C16	109.74 (12)
C8—N11—C10	106.35 (12)	C13—C18—C19	115.67 (12)
N4—N3—H42	107.00	N1—C19—C18	132.23 (13)
C5—N3—H42	129.00	C10—C19—C18	121.47 (19)
C10—N11—H43	127.00	N1—C19—C10	106.28 (15)
C8—N11—H43	127.00	C8—C20—C12	122.36 (16)
C6—C2—C10	120.30 (13)	C8—C20—C15	119.7 (2)
O9—C5—C17	129.25 (14)	O7—C22—C21	127.2 (3)
N3—C5—C17	115.3 (2)	C10—C2—H28	121.00
O9—C5—N3	115.26 (18)	C6—C13—H27	121.00
C13—C6—C14	118.42 (17)	C15—C24—C22	120.41 (18)
C2—C6—C13	118.65 (19)	C12—C21—C22	119.6 (2)
C2—C6—C14	121.70 (13)	C21—C22—C24	119.3 (3)
N11—C8—C20	125.48 (14)	C6—C2—H28	119.00
N1—C8—C20	122.63 (16)	C20—C12—H32	119.00
N1—C8—N11	111.60 (19)	C20—C15—H29	119.00
N11—C10—C19	108.49 (17)	C24—C15—H29	119.00
C2—C10—C19	120.71 (17)	C21—C12—H32	118.00
N11—C10—C2	130.76 (12)	C12—C20—C15	116.2 (3)
C20—C12—C21	122.47 (18)	C18—C13—H27	116.00
C6—C13—C18	123.12 (17)	O7—C22—C24	113.20 (18)
N4—C14—C6	115.89 (14)	C14—C16—H36	110.00
C6—C14—C16	122.25 (17)	C17—C16—H36	110.00
N4—C14—C16	121.6 (2)	C23—C16—H36	109.00
C20—C15—C24	121.7 (2)	C5—C17—H37	109.00

### Hydrogen-bond geometry (Å, º)

Cg1 is the centroid of the C12/C20/C15/C24/C22/C21 ring.

**Table:**

*D*—H···*A*	*D*—H	H···*A*	*D*···*A*	*D*—H···*A*
N3—H42···O9^i^	0.97	1.85	2.817 (3)	174
N11—H43···N1^ii^	0.95	2.27	3.2039 (19)	168
C18—H26···*Cg*1^iii^	0.97	2.43	3.369 (2)	161

Symmetry codes: (i) −*x*+1, −*y*+1, −*z*; (ii) *x*, −*y*+3/2, *z*+1/2; (iii) −*x*, *y*−1/2, −*z*−1/2.
